# O-linked *N*-acetylglucosamine affects mitochondrial homeostasis by regulating Parkin-dependent mitophagy in hyperoxia-injured alveolar type II cells injury

**DOI:** 10.1186/s12931-022-02287-0

**Published:** 2023-01-16

**Authors:** Yu Xuefei, Liu Dongyan, Li Tianming, Zheng Hejuan, Fu Jianhua

**Affiliations:** 1 Department of Pediatrics, Shengjing Hospital of China Medical University, 36 Sanhao Street, Shenyang, Liaoning 110004 People’s Republic of China; 2 Department of Gastroenterology and Medical Research Center, Liaoning Key Laboratory of Research and Application of Animal Models for Environmental and Metabolic Diseases, Shengjing Hospital of China Medical University, Shenyang, 110004 Liaoning China

**Keywords:** O-linked N-acetylglucosamine, Bronchopulmonary dysplasia, Hyperoxia, Parkin, Mitochondrial homeostasis

## Abstract

**Background:**

The level of linked N-acetylglucosamine (O-GlcNAc) has been proved to be a sensor of cell state, but its relationship with hyperoxia-induced alveolar type 2 epithelial cells injure and bronchopulmonary dysplasia (BPD) has not been clarified. In this study, we evaluated if these effects ultimately led to functional damage in hyperoxia-induced alveolar cells.

**Methods:**

We treated RLE-6TN cells at 85% hyperoxia for 0, 24 and 48 h with Thiamet G (TG), an OGA inhibitor; OSMI-1 (OS), an OGT inhibitor; or with UDP-GlcNAc, which is involved in synthesis of O-GlcNAc as a donor. The metabolic rerouting, cell viability and apoptosis resulting from the changes in O-GlcNAc glycosyltransferase levels were evaluated in RLE-6TN cells after hyperoxia exposure. We constructed rat Park2 overexpression and knockdown plasmmids for in vitro verification and Co-immunoprecipitation corroborated the binding of Parkin and O-GlcNAc. Finally, we assessed morphological detection in neonatal BPD rats with TG and OS treatment.

**Results:**

We found a decrease in O-GlcNAc content and levels of its metabolic enzymes in RLE-6TN cells under hyperoxia. However, the inhibition of OGT function with OSMI-1 ameliorated hyperoxia-induced lung epithelial cell injury, enhanced cell metabolism and viability, reduced apoptosis, and accelerated the cell proliferation. Mitochondrial homeostasis was affected by O-GlcNAc and regulated Parkin.

**Conclusion:**

The results revealed that the decreased O-GlcNAc levels and increased O-GlcNAcylation of Parkin might cause hyperoxia-induced alveolar type II cells injurys.

**Supplementary Information:**

The online version contains supplementary material available at 10.1186/s12931-022-02287-0.

## Introduction

Oxygen has a wide range of applications in saving the lives of premature infants and children with severe illnesses and respiratory failure [1]. Despite its frequent and widespread use in the clinical management of neonates to maintain blood oxygen saturation, little is known about the appropriate dose of oxygen and how long it is considered safe to use [2]. Oxygen, as an environmental stimulus, plays a crucial regulatory role in the normal development of the distal lung. Prolonged exposure to hyperoxia can alter the normal development of lung tissue and its associated vasculature and is a risk factor for bronchopulmonary dysplasia (BPD) [3–5]. During the critical period of lung development, neonatal lung hyperoxia-injured cells simultaneously undergo apoptosis and non-apoptotic cell death [6]. Increased apoptosis from hyperoxia exposure may be an important factor in impaired lung growth and remodeling, however, its underlying mechanism remains unclear.

O-linked N-acetylglucosamine (*O*-GlcNAc) levels have been shown to be sensors of cellular states (such as nutrient, stress, and cell cycle status) [7], regulating almost all cellular processes, including signaling, transcription, translation, cytoskeletal function, and cell division [8]. As an amino sugar, *O*-GlcNAc plays an important role in biological processes such as cell growth and differentiation [9, 10]. It has a unique form of glycosylation that occurs specifically on proteins in the cytoplasmic and nuclear compartments wherein it dynamically modifies the serine and threonine residues. *O*-GlcNAc transferase (OGT) adds *O*-GlcNAc to a protein via the substrate UDP-GlcNAc; the *O*-GlcNAcase (OGA) is responsible for *O*-GlcNAc removal [11]. Changes in oxygen-glucose metabolism affects the level of UDP-GlcNAc via the hexosamine biosynthesis pathway, which in turn affects the *O*-GlcNAcylation of proteins [12].

Many studies have confirmed that alveolar type II (ATII) cells are crucial to the damage and repair of lung epithelial cells [13, 14]. However, as the target cells of hyperoxia-induced lung injury, it is still unclear whether there is a change in *O*-GlcNAc levels in these cells and the underlying mechanisms. The regulation of mitochondrial homeostasis is not only crucial for scavenging reactive oxygen species (ROS) and regulating its production [15], but also an important factor affecting the quality control of lung epithelial cells in BPD [16, 17]. Due to the functions of mitochondria in cellular metabolism and regulation of different types of cell death, maintaining a functional mitochondrial network is critical for cellular homeostasis and bodily fitness in response to physiological adaptations and stressful conditions [18–21]. Therefore, we used RLE-6TN cells, a rat ATII cell line, to observe the effect of hyperoxia on cell proliferation and apoptosis, in the context of O-GlcNAc activity and mitochondrial homeostasis.

In this study, we hypothesized that O-linked N-acetylglucosamine modification is involved in Parkin-dependent pathway disorders in RLE-6TN cell lines, affecting their proliferation and apoptosis. We evaluated if these effects ultimately led to functional damage in hyperoxia-induced alveolar cells.

## Materials and methods

### Antibodies and reagents

Anti-*O*-GlcNAc monoclonal (MA1-072; Invitrogen), anti-OGT polyclonal antibody (11576–2-AP; Proteintech), anti-Oga rabbit polyclonal antibody (MGEA5 antibody, 14711–1-AP), anti-Parkin (ab15954; Abcam), anti-LC3B (2775; Cell Signalling Technology), and anti-caspase 3 monoclonal antibody (T40044; Abmart), anti-caspase 9 (9502; Cell Signalling Technology), anti-Bcl2 polyclonal antibody (26593–1-AP; Proteintech), anti-Bax polyclonal antibody (50599–1-AP; Proteintech) were used. UDP-*N*-acetyl-d-glucosamine (C3866), OSMI-1 (B7923), and Thiamet G (B2048) were purchased from APExBIO.

### Cell culture

RLE-6TN rat type II lung epithelial cells (a kind gift from MD, Jia Xianxian, Shengjing Hospital, China Medical University) were cultured in RPMI 1640 medium (Hyclone) supplemented with 10% fetal bovine serum, 100 U penicillin, and 100 mg mL^–1^ streptomycin (Sigma) at 37 °C in the presence of 5% CO_2_. Parkin (Prkn2) overexpression and knockdown plasmids were constructed and verified at Jikai Biotech. Cells were transfected using Lipofectamine 3000 (Thermo Fisher Scientific) per the manufacturer’s instructions. SiRNAs targeting *Ogt* and control siRNAs were purchased from Hanbio Biotechnology (Shanghai, China). The sequences of the rat *Ogt* siRNAs used in this study are as follows:

- control: forward, 5′-UUCUCCGAACGUGUCACGUTT-3′;
- reverse, 5′-ACGUGACACGUUCGGAGAATT-3′;
- Si01: forward, 5′-GGAAAUGUAUACAAGGAAATT-3′;
- reverse, 5′-UUUCCUUGUAUACAUUUCCTT-3′;
- Si02: forward, 5′-GCAUGUUAUUUGAAAGCAATT-3′;
- reverse, 5′-UUGCUUUCAAAUAACAUGCTT-3′;
- Si03: forward, 5′-GGAUGCUUAUAUCAAUUUATT-3′;
- reverse, 5′-UAAAUUGAUAUAAGCAUCCTT-3′;

The sequences of the plasmids (Hanbio Biotechnology) are shown in Table 1.

**Table 1:** *Park2* shRNA sequences

Name	5′	stem	loop	stem	3′
Sh-01	GATCCC	CTGGAACAACAGAGTATCGTT	CTCGAG	AACGATACTCTGTTGTTCCAG	TTTTTGGAT
Sh-02	GATCCC	AACCCTGTCTTGGTCTTCCAA	CTCGAG	TTGGAAGACCAAGACAGGGTT	TTTTTGGAT
Sh-03	GATCCC	GGCCCATCTTGCTGGGATGAT	CTCGAG	ATCATCCCAGCAAGATGGGCC	TTTTTGGAT

### Hyperoxia exposure cell model and treatment

Cells at 70–80% confluence were exposed to HO (hyperoxia) (85% O2 and 5% CO2) or NO (Normoxia) (21% O_2_ and 5% CO_2_) for 24 and 48 h. For O-GlcNAc treatment, the cells were randomly assigned to the TG group [Thiamet G (1 μmol L^–1^)]; OS group[ OSMI-1 (1 μmol L^–1^)]; UDP group[UDP-GlcNAc (20 μmol L^–1^)]; or Negative control (NC) group [(dimethyl sulfoxide; DMSO) (1 μmol L^–1^)] at 0-48 h, and culture media were changed every 24 h.

### Hyperoxia exposure animal model and treatment

Following our previously described methods [22, 23], neonatal Sprague Dawley rats in the model group were exposed to a hyperoxia environment (FiO_2_ = 0.85) within 12 h after birth, and the control group was exposed to normoxia environment (FiO_2_ = 0.21). The CO_2_ concentration was controlled at < 0.5% using soda lime, and silica gel was used to remove water vapor from the oxygen tank. The maternal rats were used to feed the neonatal rats and were exchanged among cages every 24 h to minimise the effect of differences in nursing ability. The cage was opened for 30 min everyday and clean drinking water and food were provided. The room was regulated with 12 h day/night cycle. Food and water were provided ad libitum. On postnatal day 14 after birth, 16 pup rats in each group were anaesthetised using isoflurane inhalation anesthesia and euthanized by cervical dislocation. For O-GlcNAc treatment, the rats were randomly assigned to the hyperoxia + TG group [Thiamet G (10 mg/kg per day)]; OS group [OSMI-1 (10 mg/kg per day)] or negative control group [DMSO (10 mg/kg per day)]; was intraperitoneally administrated to rats at 1–14 day.

### Western blot analysis

RLE-6TN cells were lysed using an IP lysis buffer (Sangon, Shanghai, China). Protein concentrations were determined using the bicinchoninic acid assay. Proteins were separated via 10% sodium dodecyl sulfate (SDS) polyacrylamide gel electrophoresis (separation gel: 2.5 mL acrylamide, 0.1 mL 1% SDS, 4.8 mL ddH_2_O, 0.1 mL 1% ammonium persulfate, 10 µL tetramethylethylenediamine; concentration gel: 650 µL acrylamide, 50 µL 1% SDS, 3 mL ddH_2_O, 50 µL 1% ammonium persulfate, 5 µL tetramethylethylenediamine), and then transferred to a polyvinylidene fluoride membrane (Millipore). The membrane was blocked with 5% skim milk for 120 min and incubated with specific primary antibodies [anti-*O*-GlcNAc (1:1000), anti-OGA (1:1000), anti-OGT (1:1000), and anti-β-actin (1:10,000)] at 4 °C overnight.

### Double immunofluorescence staining

AT-II cells were plated at 100,000 cells/ml and continue to incubate for 48 h in a hyperoxia or normal culture environment for subsequent detection. The plated cells were washed with PBS and fixed with 4% paraformaldehyde at 4 °C for 30 min. Primary antibodies were added, including antibodies against anti-O-GlcNAc monoclonal (MA1-072; Invitrogen), anti-OGT polyclonal antibody (11576-2-AP; Proteintech), and incubated overnight at 4 °C. After washing with PBS, donkey anti-rabbit IgG H&L (Alexa Fluor® 594; cat. no. ab150076, Abcam) and donkey anti-mouse IgG H&L (Alexa Fluor® 488; cat. no. ab150105; Abcam) were added, incubated for 2 h at room temperature, and counterstained with DAPI for 5 min at room temperature. After observation at × 400 magnification using a two-photon confocal microscope (LSM880; Zeiss AG), 3-dimensional reconstruction was performed using ImageJ software1.80, which was also used to analyse the changes in fluorescence intensity changes. Negative controls included the substitution of primary antibodies with PBS. DAPI (Sigma-Aldrich, St. Louis, MO) was used for counterstaining nuclei. Double immunofluorescence imaging was observed using a confocal laser-scanning microscope (C1, Nikon). Ten randomly selected microscopic fields of cells in each group were used to calculate the percentage of OGT-positive cells/DAPI-positive cells, O-GlcNAc-positive cells/DAPI-positive cells, and OGT&O-GlcNAc double-stained cells/DAPI-positive cells.

### Quantitative reverse transcription PCR

To quantify the gene expression, total RNA was extracted from cells using TRIzol reagent (Invitrogen, Carlsbad, USA) and cDNA was synthesized using an RT premix. Gene expression was quantified using SYBR dye and primer pairs targeting *Pink1* (F: 5′-CGAGGAGAAGCAGGCGGAGAG-3′, R: 5′-TCAGATAATCCTCCAGGCGGAAGC-3′), *Prkn (*F: 5′-TCAGAAGCAGCCAGAGGTCCAG-3′, R: 5′-GCAGGTGCCGCACTGAACTC-3′), *Ogt* (F: 5′-GAGTTGGCACATCGGGAATATCAGG-3′, R: 5′-CACCAGTATTGTCAGGCTCTTGTCTC-3′), and *Oga* (F: 5′-CCATCCACACCCTTGCCACTTG-3′, R: 5′-AGTCTCAATGTCTTCATCACTGCCTTC-3′). Relative mRNA levels were calculated using the 2^−ΔΔCT^ method based on the Ct values obtained. β-actin gene expression was measured for normalization.

### Metabolic flux analysis

Metabolic flux and oxidative phosphorylation (OXPHOS) levels were determined using a Seahorse XF96 analyzer (Agilent), following the manufacturer’s instructions. In brief, 1 × 10^5^ RLE-6TN cells/well were seeded overnight in Seahorse assay plates. Once the cells adhered to the walls of the wells, the medium was replaced with RPMI 1640 medium with 10% fetal bovine serum supplemented with 1 μmol L^–1^ Thiamet G, an Oga inhibitor; or 1 μmol L^–1^ OSMI-1, an OGT inhibitor; or 20 μmol L^–1^ UDP-GlcNAc. The cells were cultured under normoxia (21% O_2_, 5% CO_2_) or hyperoxia (85% O_2_, 5% CO_2_) for 24 h or 48 h prior to the assay. Then, the medium was replaced with Seahorse medium. Oligomycin (1 μg mL^–1^), FCCP carbonyl cyanide 4-(trifluoromethoxy)phenylhydrazone (3 μM), and a mixture of rotenone (1 μM) and antimycin A (1 μM) were added sequentially to inhibit ATP synthase, uncouple OXPHOS, and assay non-mitochondrial respiration, respectively. For the glycolysis stress test, Seahorse XF basal medium supplemented with glutamine (2 mM), 10 mM glucose, 1 μM oligomycin, and 50 mM 2-DG was used in accordance with the respective manufacturer’s instructions.

### Co-immunoprecipitation

Lung tissues and cells were lysed using IP lysis buffer (Sangon). Protein concentrations were determined using the bicinchoninic acid assay. An appropriate amount of primary antibodies (anti-*O*-GlcNAc antibody, PTM BioLabs [4 µg], Parkin antibody from Santa Cruz [4 µg], and normal mouse IgG antibody from Cell Signaling Technology [4 µg]) were added to 1000 µg of total protein and the samples were slowly shaken on a rotary shaker at 4 °C overnight. Then, 35 µL of protein A/G agarose beads (Invitrogen) was added to the samples, which were again slowly shaken on a rotary shaker at 4 °C for 2 h. Next, the beads were washed thrice with the lysis buffer, the liquid was discarded, and ddH_2_O and 4 × protein loading buffer were added to the samples, which were then heated at 100 °C for 10 min. Finally, after centrifuged at 4 °C and 1000 rpm for 1 min, the supernatants were collected and subjected to western blot analysis.

### Membrane potential measurement

Mitochondrial membrane potential was measured using 5,5,6,6′-Tetrachloro-1,1′,3,3′-tetraethylbenzimidazolylcarbocyanine chloride (JC-1) dye probe (Beyotime) according to the manufacturer’s instructions. Briefly, 20,000 cells were seeded into a 6-well plate, 2 μM JC-1 was added 48 h later, and the cells were incubated at 37 °C for another 15 min. Carbonyl cyanide chlorophenylhydrazone (CCCP) was used as a control to confirm that the JC-1 response was sensitive to changes in the membrane potential. Regions of high mitochondrial polarization are revealed by red fluorescence due to J-aggregate formation of the concentrated dye, whereas depolarized regions are indicated by green fluorescence of JC-1 monomers. Pictures are representative images of four experiments performed in live imaging condition using an inverted Leica DMi8 microscope (Leica). Results are expressed as the fluorescence intensity ratio (red/green fluorescence intensity ratio) of JC-1 staining for each image.

### Cell proliferation and cell cycle assays

Cell proliferation was assessed using a CCK-8 Cell Proliferation Assay kit (Dojindo Laboratories, Kumamoto, Japan), following the manufacturer’s instructions. RLE-6TN cells were seeded in a 96-well plate (8 replicates per condition) and treated with OSMI-1 or TG at 1 μmol L^–1^ concentrations. Then, 10 µL of CCK-8 assay reagent was added per well, and the cells were further incubated for 2 h. Finally, the absorbance of each well at 450 nm was read using an Envision plate reader (Perkin Elmer). Growth was normalized to that of untreated samples in each group.

For cell cycle analysis, cells were harvested and fixed in 70% ethanol at –20 °C for 12 h. The cells were treated with RNase A (20 mg mL^–1^) at 37 °C for 30 min, and then with 50 mg mL^–1^ propidium iodide (BD Biosciences) in the dark at room temperature for 30 min. A FACS Calibur flow cytometer (BD Biosciences) was used to assess cell cycle stage.

### Apoptosis assay

Apoptosis was assessed using a PI-FITC Apoptosis Detection Kit (BD Biosciences), as per the manufacturer’s instructions. Briefly, cells were collected using trypsin, washed with cold PBS, resuspended in 500 µL binding buffer, and incubated with 5 μL FITC and 5 μL PI in the dark for 20 min. Fluorescence signals were analyzed using flow cytometry.

### Statistical analysis

All data are expressed as the mean ± standard error of the mean (SEM). Data were analyzed using GraphPad Prism version 8.0 (GraphPad Software) or SPSS (23 IBM SPSS Statistics). The *Shapiro–Wilk* test was used to check the normality of the data sets. If the normality test failed, the data were transformed to a normal distribution. The means were compared using unpaired Student’s *t*-tests at each time point, and no multiple comparisons were made across time points or for the control vs. model at any given time point. Correlation analysis was performed using Pearson’s tests. The letter “n” represents the number of animals in each group or the number of independent experiments. To compare multiple means, we used two-way ANOVA. The degrees of freedom for all repeated measures ANOVAs were adjusted using Greenhouse–Geisser correction. For all comparisons, *P* < 0.05 was considered significant.

## Results

### The expression of *O*-GlcNAc and its metabolic enzymes were downregulated in hyperoxia-induced RLE-6TN cells injure

**Fig. 1:**
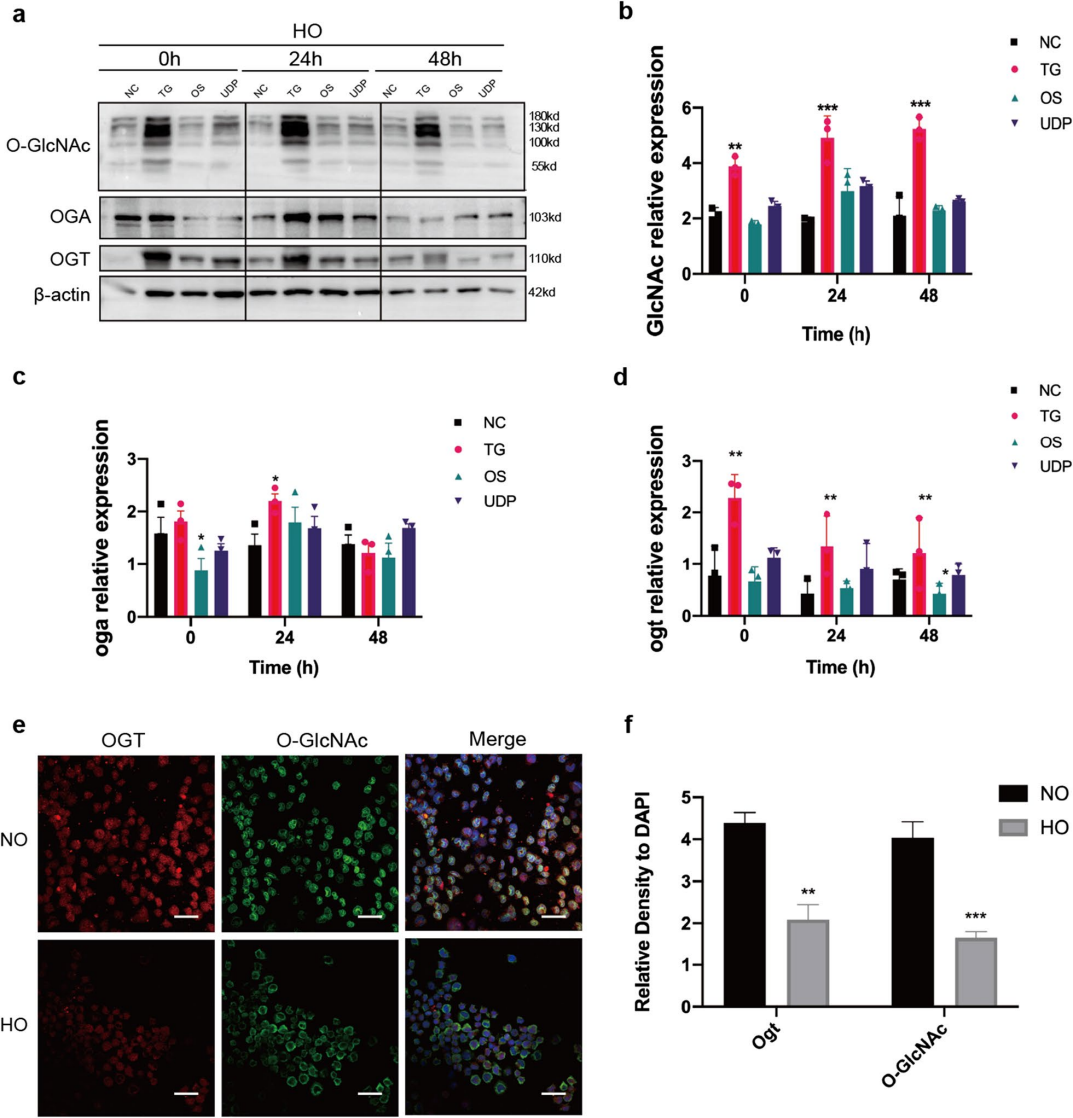
The expression of *O*-GlcNAc and its metabolic enzymes were downregulated in hyperoxia-exposed RLE-6TN cells. **a** Western blot analysis of *O*-GlcNAc, OGA, and OGT levels in hyperoxia-exposed RLE-6TN cells. **b**–**d** Gray value analysis of the western blot of *O*-GlcNAc, OGA, and OGT expression based on **a**. **e** Immunofluorescence detection of *O*-GlcNAc expression in RLE-6TN cells after hyperoxia (magnification × 400; scale bar = 50 µm). **f** Semi-quantitative analysis of immunohistochemistry based on immunofluorescence. Data are shown as the mean ± SEM (n = 3) and were analyzed using two-way ANOVA followed by Tukey’s post-hoc tests. *P < 0.05, **P < 0.01, ***P < 0.001 compared to the negative control (dimethyl sulfoxide; DMSO) group; NO: normal air-exposed group; HO: hyperoxia-exposed group. NC: negative control group; TG: Thiamet G-treated group; OS: OSMI-1-treated group; UDP: UDP-GlcNAc-treated group

The western blot results indicated that after TG and UDP-GlcNAc treatment, the level of *O*-GlcNAc was significantly higher. But the expression levels of OGT, OGA and *O*-GlcNAc did not change significantly in RLE-6TN cells after exposure to high levels of oxygen (Fig. 1a–d). The results of the immunofluorescence assay showed that OGT and *O*-GlcNAc content decreased after 48 h of exposure to high oxygen levels (Fig. 1e–f).

### OSMI-1-induced inhibition of OGT and increased the proliferation of hyperoxia-induced RLE-6TN cells injures

**Fig. 2:**
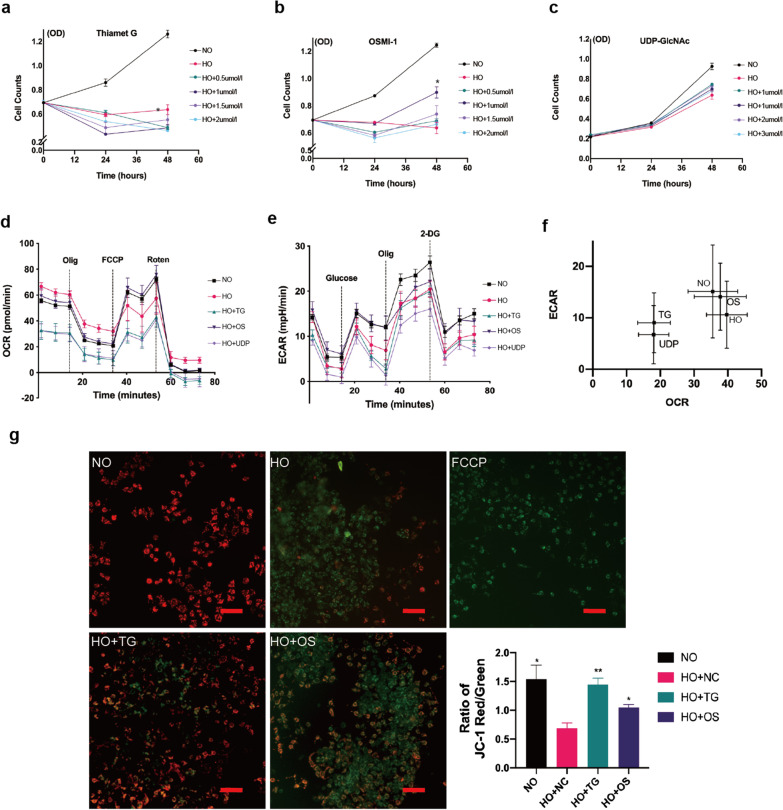
Interference with *O*-GlcNAc metabolic enzymes modulated mitochondrial metabolic function and affects mitophagy in hyperoxia-exposed RLE-6TN cells. **a** CCK-8 assay of cell proliferation after exposing RLE-6TN cells to hyperoxia and treatment with 1 μmol L^–1^ Thiamet G. **b** CCK-8 assay of cell proliferation after exposing RLE-6TN cells to hyperoxia and treatment with 1 μmol L^–1^ OSMI-1. **c** CCK-8 assay of cell proliferation after exposing RLE-6TN cells to hyperoxia and treatment with 20 μmol L^–1^ UDP-GlcNAc. **d–f** Metabolic profiles determined using extracellular flux analysis (Seahorse XF). The oxygen consumption rate (OCR) and extracellular acidification rate (ECAR) in each condition are shown, following the addition of oligomycin (0.5 μM), FCCP (0.25 μM), and antimycin (1 μM) and rotenone (1 μM), glutamine (2 mM), 10 mM glucose, 1 μM oligomycin, and 50 mM 2-DG according to the protocol. **g** Graphs of JC-1 fluorescence as a measure of the cell membrane potential. Red indicates a normal membrane potential, whereas green indicates a reduced membrane potential (magnification × 400; scale bar = 50 µm). Data are shown as the mean ± SEM (n = 6) and were analyzed using two-way ANOVA followed by Tukey’s post-hoc tests. *P < 0.05, **P < 0.01. NO: normal air-exposed group; HO: hyperoxia-exposed group. HO + TG: 48 h hyperoxia-exposed and Thiamet G-treated group; HO + OS: 48 h hyperoxia-exposed and OSMI-1-treated group; UDP: 48 h hyperoxia-exposed and UDP-GlcNAc-treated group; FCCP: carbonyl cyanide 4-(trifluoromethoxy)phenylhydrazone

We used the CCK-8 assay to evaluate the effect of the treatments on cell proliferation under hyperoxia exposure. After 24 h of hyperoxia, the number of cells decreased by 31.0% compared with group NO; after 48 h, the number of cells decreased by 49.5% compared with group NO (*P* < 0.05). Cell proliferation decreased by 49.6% after 24 h hyperoxia exposure and treatment with 1 μmol L ^–1^ Thiamet G and 61.5% after 48 h exposure to hyperoxia alone (*P* < 0.05). In contrast, treatment with OSMI-1 for 48 h (increased by 41.1% compared with group HO) or UDP-GlcNAc (increased by 19.3% compared with group HO) improved the proliferation of lung epithelial cells after 48 h of hyperoxia exposure (Fig. 2a–c). It is suggested that the inhibition of OGT can alleviate hyperoxia injury.

### Downregulation of *O*-GlcNAc metabolic enzymes modulated the mitochondrial metabolic homeostasis in hyperoxia-induced RLE-6TN cells injures

Next, we evaluated the metabolic rerouting resulting from the changes in *O*-GlcNAc glycosyltransferase levels in RLE-6TN cells after hyperoxia exposure as a real-time measurement of oxygen consumption rate (OCR, an OXPHOS indicator) (Fig. 2d) and extracellular acidification rate (ECAR, used for aerobic glycolysis) (Fig. 2e).

Compared with the control group, the mitochondrial stress and basic mitochondrial respiratory capacity increased after 48 h of exposure to high levels of oxygen, but the maximum mitochondrial respiratory capacity decreased. The mitochondrial metabolism level of RLE-6TN cells increased after OSMI-1 treatment upon exposure to high oxygen levels. There was no significant difference in basal respiration between RLE-6TN cells in the OSMI-1 group, but the respiratory reserve capacity was significantly increased (*P* < 0.05). Maximum respiration increased by 32.0% in the OSMI-1-treated group compared with the high oxygen exposure group. However, the levels of mitochondrial stress and aerobic metabolism decreased after 48 h hyperoxia exposure and Thiamet G or UDP-GlcNAc treatment (25.2% and 28.4% in the TG and UDP groups, respectively). Compared with the control group, the anaerobic acidification rate, glycolysis capacity, and glycolysis reserve capacity of RLE-6TN cells decreased (*P* < 0.05) after 48 h of exposure to high oxygen levels. OSMI-1 treatment increased glycolysis capacity but not glycolysis reserve capacity. The glycolytic ability of cells in the Thiamet G and UDP-GlcNAc groups decreased. The overall energy distribution suggested that the changes in the levels of *O*-GlcNAc metabolic enzymes affected the metabolic ability of RLE-6TN cells (Fig. 2f).

The use of JC-1 to evaluate mitochondrial membrane potential (MMP) showed that the reduction of MMP in hyperoxia-exposed lung epithelial cell line was relieved when treated with TG. (Fig. 2g). Together, these results indicated that interference with *O*-GlcNAc enzymes could affect mitochondrial metabolic function and homeostasis of RLE-6TN cells under hyperoxic conditions.

### *O*-GlcNAc affects mitochondrial homeostasis by regulating Parkin-dependent mitophagy

**Fig. 3:**
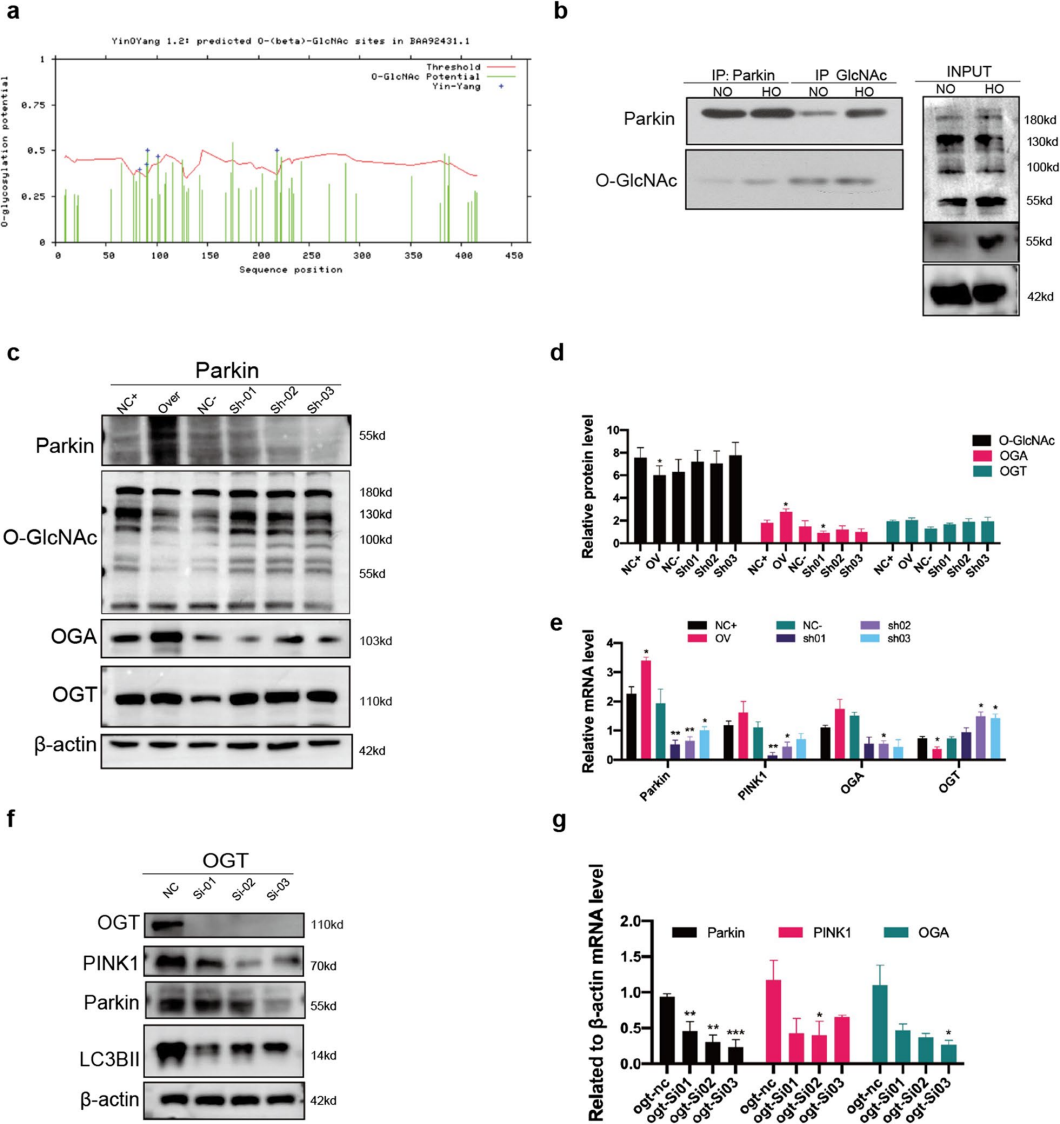
OGT increased the expression of Pink1/Parkin, and Parkin might play a negative feedback role on OGT in RLE-6TN cells. **a** Prediction of potential *O*-GlcNAcylation sites in rat Parkin protein using YinOYang (http://www.cbs.dtu.dk/services/YinOYang/). **b** Co-immunoprecipitation analysis of the binding between *O-*GlcNAc and Parkin. **c** Western blot analysis of *O-*GlcNAc, OGA, OGT, and β-actin in *Parkin*-overexpression and -knockdown RLE-6TN cells. **d** Gray value analysis of the western blot of *O*-GlcNAc, OGA, and OGT in *Parkin*-overexpression and -knockdown RLE-6TN cells. **e** qPCR analysis of *O-*GlcNAc, *Oga*, and *Ogt* levels in *Park2*-overexpression and -knockdown RLE-6TN cells. **f** Western blot analysis of OGT, Pink1, Parkin, LC3BII, and β-actin in *Ogt*-knockdown RLE-6TN cells. **g** qPCR analysis of *Parkin*, *Oga*, and *Bax* expression after *Ogt* knockdown in RLE-6TN cells. Data are shown as the mean ± SEM (n = 6) and were analyzed using two-way ANOVA followed by Tukey post-hoc tests. *P < 0.05, **P < 0.01. NC: Negative control; OV: overexpression; Sh RNA: short hairpin RNA; Si RNA: short interfering RNA

We used YinOYang (http://www.cbs.dtu.dk/services/YinOYang/) to predict potential *O*-GlcNAc modification sites in rat Parkin (Fig. 3a). We found that the mitochondrial autophagy-related protein Parkin may have *O*-GlcNAc modifications at Thr-91 and Ser-218 (*P* < 0.05). Co-immunoprecipitation corroborated the binding of Parkin and *O*-GlcNAc. The results showed that Parkin *O*-GlcNAcylation was increased in rat lung tissues after hyperoxia (Fig. 3b). To gain insight into the underlying mechanism, we constructed rat *Park2* overexpression and knockdown plasmids for in vitro verification. At the protein level, compared with the control group, *O*-GlcNAc levels in the Parkin-overexpressing group were decreased, whereas the *O*-GlcNAc levels in the knockdown group were increased. OGT expression was increased in the Parkin-overexpression group and decreased in the knockdown group (*P* < 0.05). The results showed that after affecting Parkin levels, the level of *Oga* was consistent with that of Parkin, whereas that of *Ogt* showed the opposite trend (*P* < 0.05) (Fig. 3c–e).

According to the above results, an elevation in Parkin expression can positively regulate OGA levels and negatively regulate OGT levels. Next, we used small interfering RNAs (siRNAs) targeting *Ogt* to interfere with GlcNAc transfer. The expression levels of Pink1 and Parkin decreased after *Ogt* knockdown (Fig. 3f–g). Based on the above results, we speculated that *O*-GlcNAc levels could positively regulate the expression of mitochondrial autophagy-related proteins Pink1/Parkin, and that Parkin protein might play a negative feedback role on *O*-GlcNAc transfer.

### OGT affects the mitochondrial apoptosis pathway in hyperoxia-exposed RLE-6TN cells

**Fig. 4:**
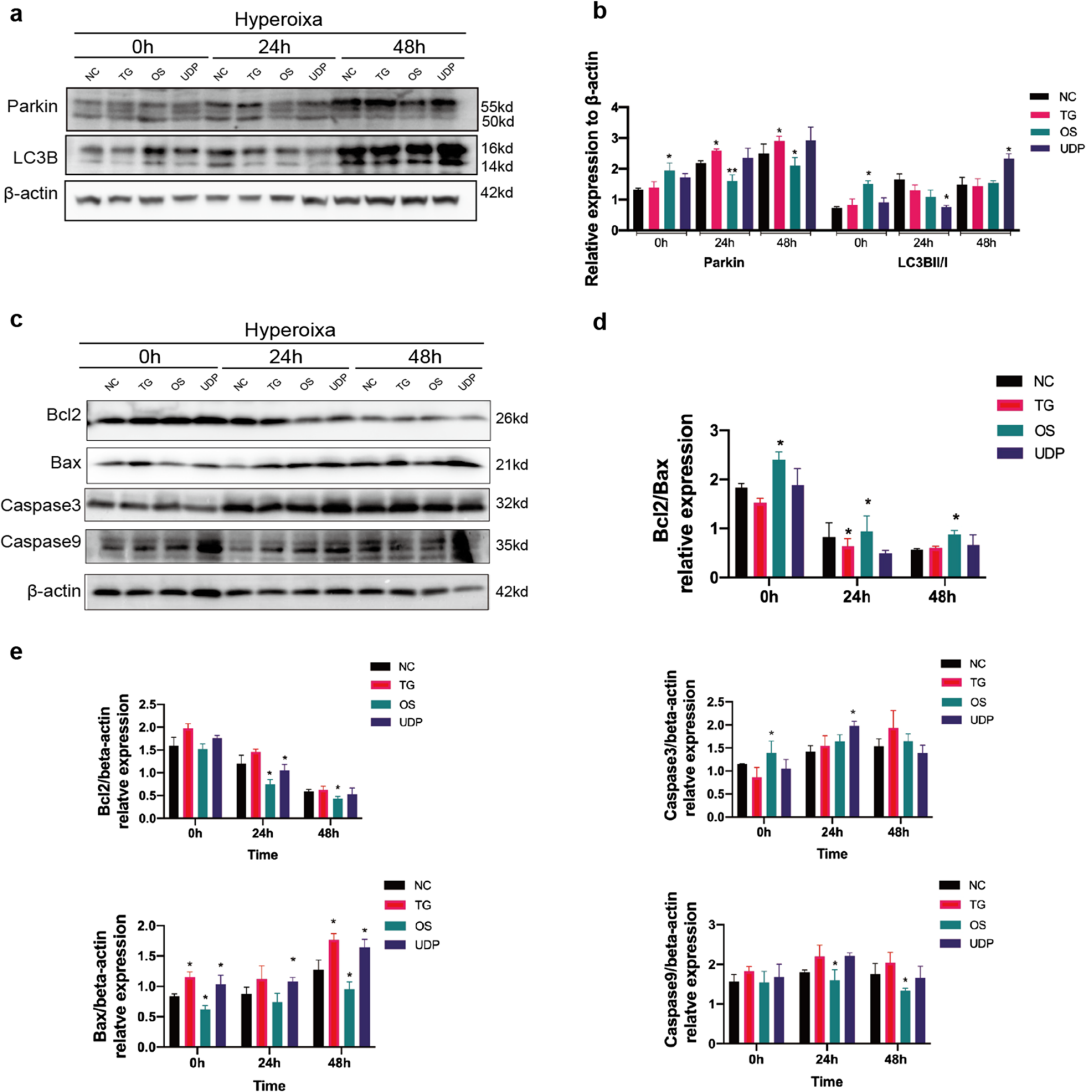
Effect of *O*-GlcNAc on the apoptosis of RLE-6TN cells induced by hyperoxia. **a** Western blot analysis of Parkin and LC3B expression in RLE-6TN lung epithelial cells after exposure to hyperoxia. **b** Quantification of Parkin and LC3B levels based on (**a**). **c** Western blot analysis of Bcl2, Bax, caspase 3 and caspase 9 expression in RLE-6TN lung epithelial cells after exposure to hyperoxia. d The Bcl2/Bax expression ratio based on (**c**). **e** Quantification of Bcl2, Bax, caspase 3 and caspase 9 levels based on (**c**). Data are shown as the mean ± SEM (n = 6) and were analyzed using two-way ANOVA followed by Tukey’s post-hoc tests. *P < 0.05, **P < 0.01. NO: normal air-exposed group; HO: hyperoxia-exposed group. NC: negative control group; TG: Thiamet G-treated group; OS: OSMI-1-treated group; UDP: UDP-GlcNAc-treated group

Some studies suggest that Pink1/Parkin-mediated mitophagy is closely related to cell death and apoptosis [24, 25]. In this study, we found that the expression of Parkin and LC3B in alveolar epithelial cells exposed to hyperoxia was increased. In addition, TG and UDP positively regulated the expression of Parkin and LC3B, whereas OSMI-1 negatively regulated their expression (Fig. 4a, b). The anti-apoptotic protein Bcl2 can preserve membrane potential [26], whereas pro-apoptotic proteins such as Bax abolish the mitochondrial membrane potential by affecting PTP, promoting downstream of the apoptotic pathway [27]. In this study, the expressions of apoptosis-related proteins Bax, caspase 3, and caspase 9, were increased in epithelial cells exposed to hyperoxia. Moreover, the Bcl2/Bax expression ratio decreased continuously after hyperoxia exposure for 48 h (*P* < 0.05). TG and UDP treatment induced the expression of caspase 3, and caspase 9. In contrast, OSMI-1 increased the Bcl2/Bax expression ratio and decreased the expression of caspase 3, and caspase 9 in hyperoxia-damaged RLE-6TN cells (Fig. 4c–e).

### *O*-GlcNAc enzyme activity affects the cell cycle in hyperoxia-exposed RLE-6TN cells

**Fig. 5:**
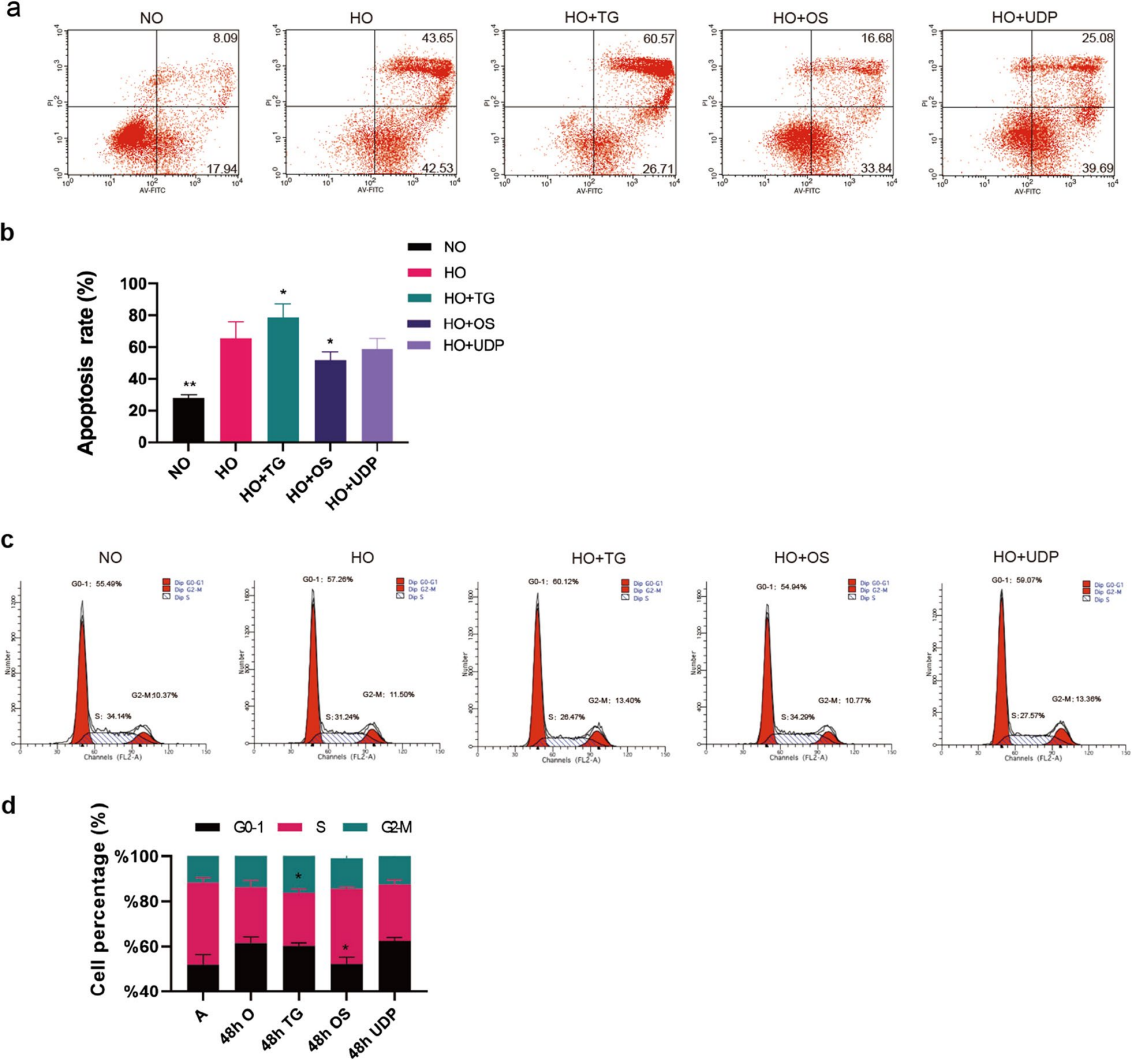
Effect of *O*-GlcNAc on apoptosis of RLE-6TN cells induced by hyperoxia. **a** Effect of *O*-GlcNAc transferase inhibitors on RLE-6TN cell apoptosis after hyperoxia exposure, as estimated via PI-FITC staining and flow cytometry. **b** Apoptosis rate of cells treated with different inhibitors of *O*-GlcNAc transferase after hyperoxia exposure. **c** Cell cycle distribution as measured via PI staining and flow cytometry. The values are expressed as the ratio of cells in the G0-G1, S, and G2-M phases after treatment with Thiamet G, OSMI-1 and UDP-GlcNAc. **d** The proportion of cells in each stage of the cell cycle in different groups after hyperoxia exposure (Data are shown as the mean ± SEM (n = 6) and were analyzed using two-way ANOVA followed by Tukey’s post-hoc tests. *P < 0.05, **P < 0.01. NO: normal air-exposed group; HO: hyperoxia-exposed group. HO + TG: 48 h hyperoxia-exposed and Thiamet G-treated group; HO + OS: 48 h hyperoxia-exposed and OSMI-1-treated group; HO + UDP: 48 h hyperoxia-exposed and UDP-GlcNAc-treated group

Apoptosis analysis via flow cytometry revealed that after 48 h of exposure to hyperoxia, Thiamet G treatment increased the fraction of RLE-6TN cells in apoptosis, whereas OSMI-1 treatment decreased the fraction of cells in late apoptosis and overall apoptosis levels (Fig. 5a, b). We then evaluated via flow cytometry whether *O*-GlcNAc affects cell cycle regulation in RLE-6TN cells. The results showed that the percentage of cells in the G0-1 phase increased after oxygen exposure, whereas the number of cells in the S phase decreased. After OSMI-1 treatment, a larger proportion of cells remained in the S phase (Fig. 5c–d). Together, these results indicated that *O*-GlcNAc is involved in cell cycle control in hyperoxia-induced ATII injury and that the blockade of OGT suppresses apoptosis and speeds up the cell cycle.

### Effect of O-GlcNAc enzyme inhibitor in alveolarization of hyperoxia-exposed neonatal rats

**Fig. 6:**
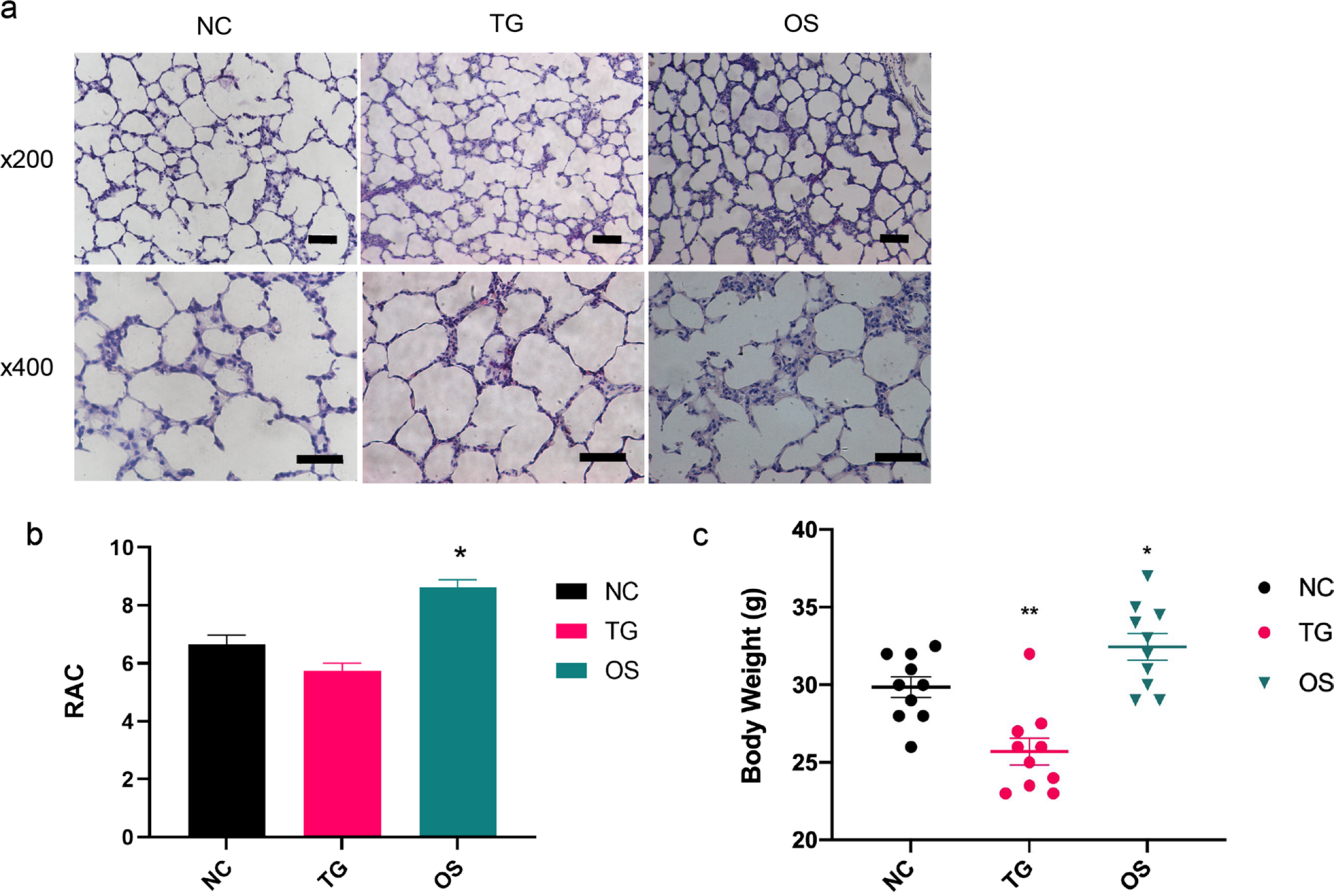
Effect of O-GlcNAc enzyme inhibitor in alveolarization of hyperoxia-exposed neonatal rats. **a** Hematoxylin–eosin staining (H&E) for histopathological observation of alveolar morphology (scale bars = 50 μm). **b** Radial alveolar counts (RAC) values in groups determined by measuring the vertical distance from the center of a bronchiole to the nearest fibrous septum or pleura. **c** Body weight at 14 days of each group. Data are shown as the mean ± SEM (n = 10) and were analyzed using two-way ANOVA followed by Tukey’s post-hoc tests. *P < 0.05, **P < 0.01. NO: normal air-exposed group; HO: hyperoxia-exposed group. NC: negative control group; TG: Thiamet G-treated group; OS: OSMI-1-treated group; UDP: UDP-GlcNAc-treated group

To evaluate the effect of O-GlcNAc enzymatic on alveolarization in neonatal rats, we administered daily doses to rats until the 14th day after birth and measured radial alveolar counts (RAC). The results of alveolar development were assessed using H&E staining and the OSMI-1 group promoted alveolarization and increased RAC rate compared with the control group, while Thiamet G group aggravated the simplification of alveolar structure (Fig. 6a). The rats housed in a hyperoxia environment and treated with OSMI-1 exhibited significantly higher RAC values than rats in hyperoxia and treated with the control group (Fig. 6b). The body weights were comparable among groups at birth, decreased in Thiamet G groups and increased in OSMI-1 groups after 14 days (Fig. 6c). These results validated that inhibition of OGT promotes hyperoxia-induced alveolar simplification in neonatal rats.

## Discussion

**Fig. 7:**
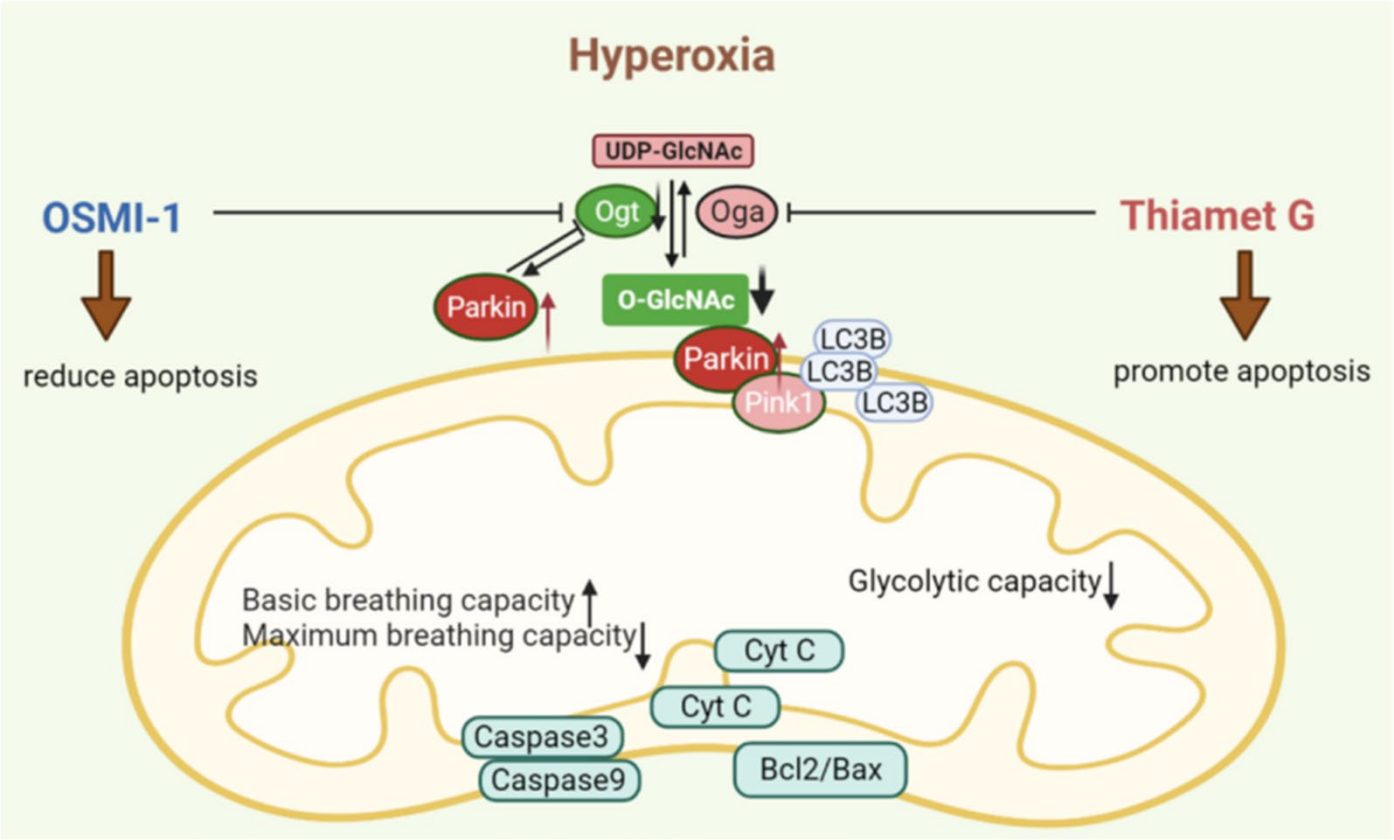
Diagrammatic representation. In this study, we proposed that the level of O-GlcNAc glycosylation affected mitochondrial metabolism and mitochondrial function after hyperoxia exposure in AT2 cells line. O-linked N-acetylglucosamine modification is involved in Parkin-dependent pathway disorders in RLE-6TN cell lines, affecting their proliferation and apoptosis

Diseases such as idiopathic pulmonary fibrosis, chronic obstructive pulmonary disease, and bronchopulmonary dysplasia injure the gas-exchanging alveoli of the human lung [28]. In neonatal intensive care, oxygen is used to treat infants who are not about to breathe oxygen independently. However, inappropriate long-term exposure to hyperoxia factors that interfere with lung developmental programs may lead to stagnation of lung development and increase the risk of developing respiratory diseases later in life [29]. ATII cells are a key structure of the distal lung epithelium, where they exert their innate immune response and serve as progenitors of alveolar type I (ATI) cells, contributing to alveolar epithelial repair and regeneration [30]. But the pathological mechanism of the damage and repair process when exposed to hyperoxia is still unclear [31, 32]. Therefore, in this study, we explored the molecular mechanism of ATII cells injury induced by hyperoxia exposure (Fig. 7).

In this study, it was confirmed that *O*-GlcNAc affects cell function, further affecting the pathological outcome of RLE-6TN cells by regulating mitochondrial homeostasis. Long-term exposure to hyperoxia arrests the development of alveolar structure and decreases the expression of OGT and the production of GlcNAc. Furthermore, we discovered that changes in *O*-GlcNAc levels might affect mitochondrial homeostasis and metabolic function by affecting Parkin-mediated autophagy pathways. Recent studies have found that *O*-GlcNAc can regulate autophagy [33–35], but related research on its underlying mechanisms is still lacking. OGT downregulation can induce cisplatin resistance in ovarian cancer by promoting autophagy [36]. OGT also affects mitochondrial mass through Pink1-dependent mitochondrial phagocytosis-mediated dysregulation of H3K4me3, regulating hematopoietic stem cell maintenance and stress responses [37]. *O*-GlcNAc inhibition significantly enhances autophagic flux, whereas autophagic flux decreases at high levels of *O*-GlcNAc. The phosphorylation of AMPK was also found to be increased upon inhibition of O-GlcNAc modification, and thereby inhibiting ULK1 activity and autophagy [38]. OSMI-1 is believed to inhibit O-linked N-acetylglucosamine in cells without qualitatively changing the N- or O-linked glycans on the cell surface. Studies have shown that OSMI-1 can promote tumor cell apoptosis and has certain prospects for cancer treatment [39, 40]. It has been shown to be negatively regulated in osteoclasts [41] and positively regulated during embryonic differentiation [42] and in corneal epithelial cells [43]. In this study, using in vitro experiments, we proved that the application of OSMI-1 to inhibit the function of OGT can improve the degree of damage to lung epithelial cells caused by hyperoxia, enhance cell metabolism and proliferation, decrease apoptosis, and promote cell cycle rates.

We further found that the mitochondrial membrane potential increased after OSMI-1 treatment. The mitochondria play an important role in regulating energy metabolism and processes associated with cell death and survival [44]. The expression of apoptosis-related proteins in epithelial cells under hyperoxia was detected by western blotting. In vitro, TG and UDP treatment increased the expression of caspase 3, and caspase 9; OSMI-1 decreased the expression of Bcl2/Bax in hyperoxia-injured RLE-6TN cells. The results of flow cytometry detection showed that Thiamet G treatment increased the proportion of RLE-6TN cells in late apoptosis after 48 h of hyperoxia exposure, whereas OSMI-1 treatment decreased the proportion of RLE-6TN cells in late apoptosis and the overall proportion of apoptotic cells. We then assessed via flow cytometry whether *O*-GlcNAc affected cycle regulation in RLE-6TN cells. The results showed that the proportion of cells in the G0-1 phase increased and the proportion of cells in the S phase decreased after the oxygen exposure. After OSMI-1 treatment, more cells stayed in S phase. Taken together, these results suggest that *O*-GlcNAc is involved in cell cycle control in hyperoxia-induced injurys and that blocking OGT can inhibit apoptosis and accelerate the cell cycle.

Moreover, we verified that there is an increase in the levels of *O*-GlcNAc-modified Parkin in alveolar cells exposed to hyperoxia and that the mitophagy pathway mediated by Parkin affects cell apoptosis and the cell cycle. A limitation of this study is that we have not clearly verify the modifications at Thr-91 or Ser-218. However, through immunoprecipitation, we initially verified that *O*-GlcNAc-modified Parkin play an important role in BPD. This result lays the foundation for future research in this process. Given that the Parkin pathway plays a key role in energy metabolism, oxidative stress, inflammation, and apoptosis [45–47]. O-GlcNAc may affect hyperoxia-induced reprogramming of the lung mitochondrial phenotype by modifying Parkin, which may have important implications for lung pathophysiology and developmental outcomes (Additional file 1).

In conclusion, this study demonstrated that the exposure of lung epithelial cells to hyperoxia may affect *O*-GlcNAc levels, ultimately affecting apoptosis and the cell cycle by mediating Parkin signaling. Further elucidation of the molecular mechanism underlying hyperoxia-induced lung injury may help develop methods to target and prevent ATII cell damage in the future.

## Supplementary Information

**Additional file 1.** For Thiamet G and OSMI-1 treatment, the cell viability of different dose was measured in the pre-experiments. a The cycle diagram of O-GlcNAc. b The cell viability was the highest at 1 μmol/l of TG. c The cell viability was the highest at 1 μmol/l of OSMI-1.

## Data Availability

The data that support the findings of this study are available from the corresponding author upon reasonable request.
