# Supplementary material for: O-linked N-acetylglucosamine affects mitochondrial homeostasis by regulating Parkin-dependent mitophagy in hyperoxia-injured alveolar type II cells injury
Source: Respir Res. 2023 Jan 16;24:16. doi: 10.1186/s12931-022-02287-0 (PMC9841680; doi:10.1186/s12931-022-02287-0)

## Additional files 1. **For Thiamet G and OSMI-1 treatment, the cell viability of different dose was measured in the pre-experiments.** a. The cycle diagram of O-GlcNAc. b. The cell viability was the highest at 1μmol/l of TG. c. The cell viability was the highest at 1μmol/l of OSMI-1.


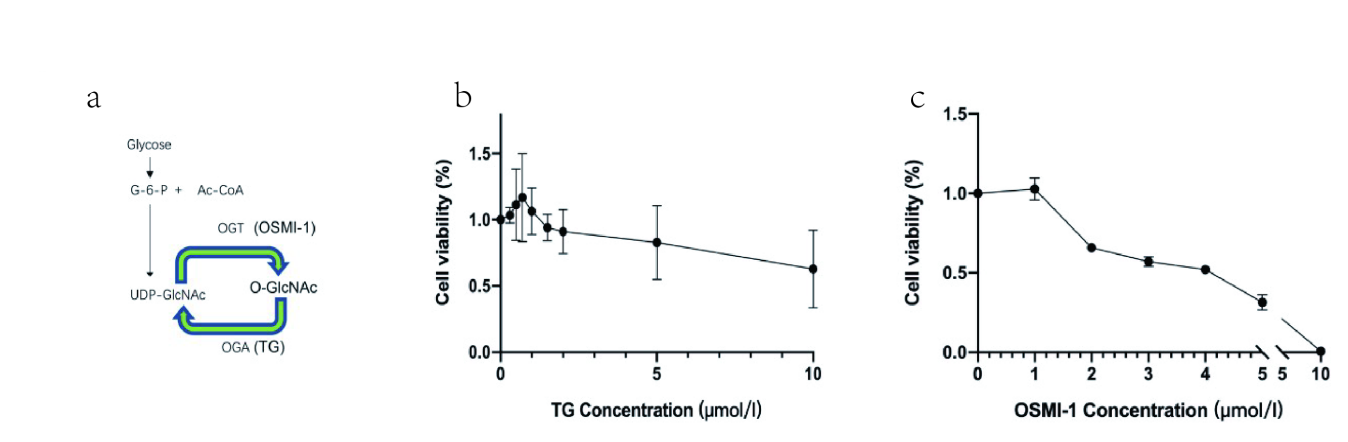

Supplement: Supplementary file 1 — Additional file 1. For Thiamet G and OSMI-1 treatment, the cell viability of different dose was measured in the pre-experiments. a The cycle diagram of O-GlcNAc. b The cell viability was the highest at 1 μmol/l of TG. c The cell viability was the highest at 1 μmol/l of OSMI-1. [file 12931_2022_2287_MOESM1_ESM.docx]
